# *Global Health Action*: surviving infancy and taking first steps

**DOI:** 10.3402/gha.v6i0.22815

**Published:** 2013-09-20

**Authors:** Nawi Ng, Peter Byass, Stig Wall, for the Editorial Team of Global Health Action

In this fifth anniversary editorial for *Global Health Action (GHA)*, we share with our readers and authors our reflections on the positive development of our journal during its first five years. At the same time, we wish to acknowledge the high-quality work of our many reviewers and the commitment of our editorial board members and mentors.

## Locating *GHA* to address the digital divide in global health research

*GHA* was launched at the end of May 2008 as a collaborative effort between Umeå Centre for Global Health Research at Umeå University and Co-Action Publishing. The journal was established to ‘contribute to fuelling a more concrete, hands-on approach to global health challenge’, and to address the global health agenda, with a strong focus on policy and implementation (1). *GHA* welcomes papers on strengthening health information, understanding health determinants in cross-cultural settings, documenting effective health interventions in local settings, and contributing to a better understanding of the performance of health systems in different settings. *GHA* calls for a strong emphasis on action, previously not addressed strongly by journals in the field of global health (1, 2).

**Fig. 1:**
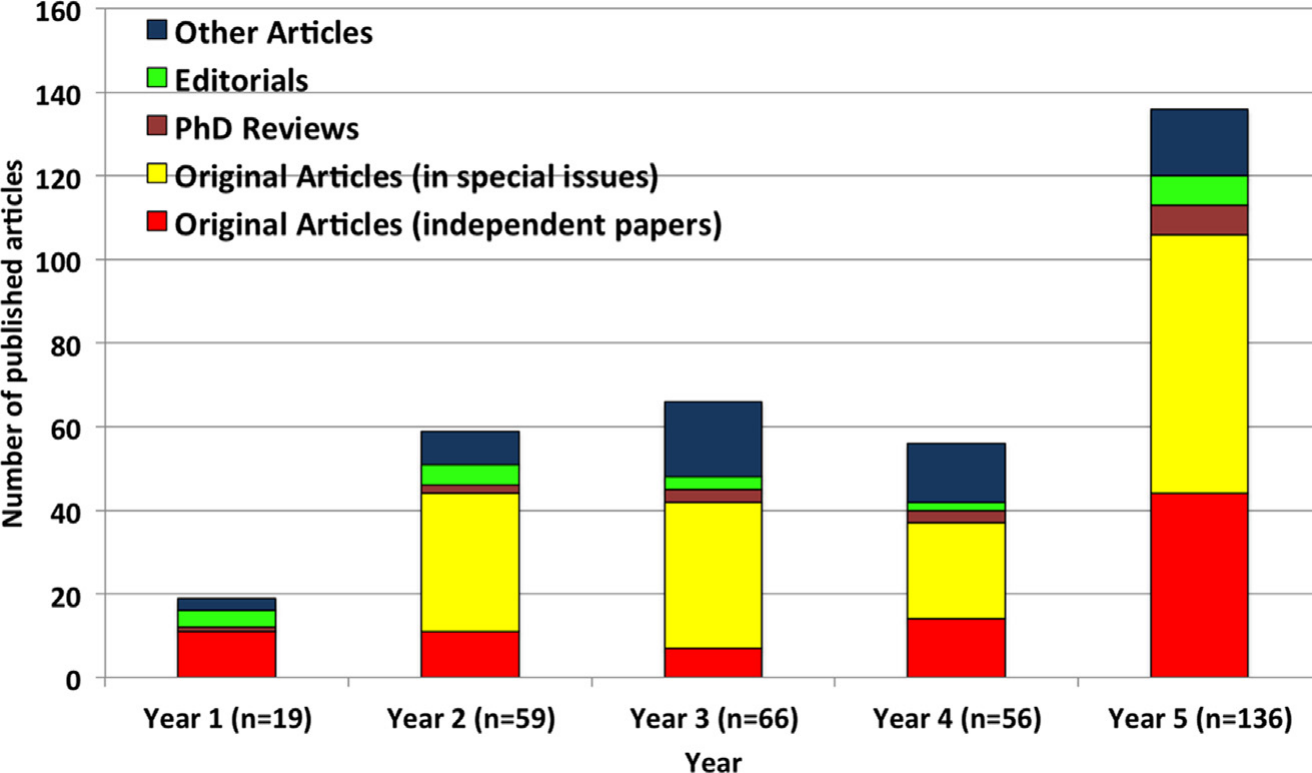
Types of papers published in *Global Health Action* (May 2008–June 2013).

During its first five years (May 2008–June 2013), *GHA* has published 336 articles (which include 87 original articles published as independent papers, 153 original articles in special issues, 21 editorials, 16 PhD reviews, and 59 articles of other types). Contributions are from 1,423 authors (of which, 919 are unique authors) from about 220 cities in 58 countries in the world. The number of papers published in *GHA* increased from 19 papers during the first year (May 2008–June 2009) to 136 papers in the fifth year (July 2012–June 2013). Figure 1 shows the number of different types of papers published in *GHA* during May 2008–June 2013.

**Fig. 2:**
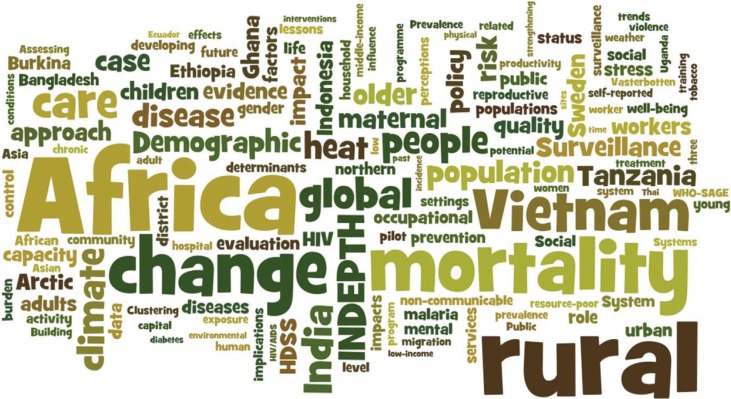
Word clouds from titles of articles published in *Global Health Action* 2008–2013. The figure was created in Wordle™ (www.wordle.net).

The articles published in *GHA* represent broad areas of global health research ranging from mortality, climate change, demography and health systems operation and financing to disease and risk factor surveillance, with the vast majority of research being conducted in Africa, Asia, and in rural settings, where high-quality research evidence has been generally lacking. Figure 2 shows a ‘word cloud’ based on the titles of all articles published in *GHA* during the first five years.

## Addressing key niches and taking innovative action

During the first five years, we focused on three niches which differentiate us from the other journals in the same field: *mentorship* to young researchers from low- and middle-income countries (LMICs); *PhD review* papers which help young researchers to kick start and sustain their research careers; and *study design* and *capacity building* articles to document details of research design and capacity building activities in LMICs.

### Mentorship

High-quality research evidence from LMICs is often lacking and inaccessible to the global scientific community. While epidemiological citations in PubMed from many African and Asian countries occur at a rate of less than 0.05 per 1,000 populations, the rate is over 1 per 1,000 in Scandinavia and elsewhere, that is, 20-fold greater (3). The immediate consequence of this is that health policy and planning in many countries, as well as at a global level, continues to struggle with a severely inadequate evidence base, and in some cases, scarce health resources are not used effectively because of this lack of evidence.

This phenomenon is due not so much to a lack of quality of the research generated, but because of a lack of capacity to report the results according to the rigorous standards required for international scientific publication. A substantial amount of effort is required to raise a poorly presented, but promising, paper to international standards. This goes way beyond the support that can reasonably be given by reviewers and editors, which is why such papers usually end up being rejected. *GHA* is highly committed to building research capacity globally by offering academic mentorship to less-experienced researchers in order to help in the development of high-quality manuscripts. *GHA* has been successful in providing the additional level of support needed by researchers in their early careers. A group of multidisciplinary and experienced researchers in global health have committed themselves to serve as such mentors for *GHA*, who are recognized in the published articles as ‘Contributing Editors’, should the paper be accepted after the peer review process. One example of such mentorship can be shown in the supplement ‘Public health in Vietnam: here's the data, where's the action?’ where three guest editors acted as mentors and built capacity among local researchers in Vietnam to write and publish high-quality scientific papers. This mentorship was built on top of the regular peer review process followed by *GHA*.

### PhD review papers

*PhD review papers* are a special niche that was initiated by *GHA* in 2009. Young researchers who have recently defended their PhD within the field of global health are invited to submit a paper based on their PhD. One rationale for this is that PhD theses are often based on a set of published articles synthesized into a ‘cover story’ of some 30–50 pages. Some of these syntheses provide excellent reviews of the research area but they seldom reach beyond the host institution or the close collaborators and examiners of the student. Condensing this content into a PhD review paper may serve as an incentive for a young researcher to publish his or her first postdoc paper as a sole author. Should the paper be accepted after peer review, the opponent or examiner is invited to write a commentary, for publication alongside the PhD review.

An example of a PhD review paper is that of Mark Collinsson, based on his PhD thesis “Striving against adversity: the dynamics of migration, health and poverty in rural South Africa” published in 2010 (4) along with a commentary by the opponent Professor Kim Streatfield (5). *GHA* readers can also download a PowerPoint file from the field site Agincourt in South Africa, as well as a video showing the public defence on 15 May 2009, from the *GHA* website.

### Capacity building and study design papers

Capacity building articles are published to document how intervention strategies in dealing with major public health issues have been developed, sometimes translated, to fit different contextual settings in LMICs. These papers reflect how partnerships and collaboration are established and built over time, and how research capacities are being built and sustained in LMICs. One example is a recently published capacity building article that dissects North-South partnerships for health in Tanzania (6). This article discusses the process and impact of scaling up on Southern partners’ organizational functioning on HIV and AIDS issues in the Kilimanjaro Region in Tanzania.

Study design articles are published as a response to the need for long-term epidemiological studies and multicentre studies to be able to set the scene for international collaborations in a methodological paper presenting the design, rationale and aims as well as hypotheses and background data to be referenced in forthcoming more detailed papers.

One example is a recently published design article from the ‘DengueTools’ research project, which is funded under the health theme of the 7th Framework Programme of the European Community. This article reports on the rationale and specific study objectives of the project, which is a consortium of 14 partners from several countries in Europe, Asia, and South America (7). Another study design article outlines the role that members of the Health and Demographic Surveillance Systems (HDSSs) of the INDEPTH Network could play in monitoring progress towards achieving the Millennium Development Goals (MDGs) (8). The unique qualities of the data generated by HDSSs lie in the fact that they provide an opportunity to measure or evaluate interventions longitudinally, through a long-term follow-up of defined populations.

## Gaining trust from authors

### Submission and rejection rate

**Fig. 3:**
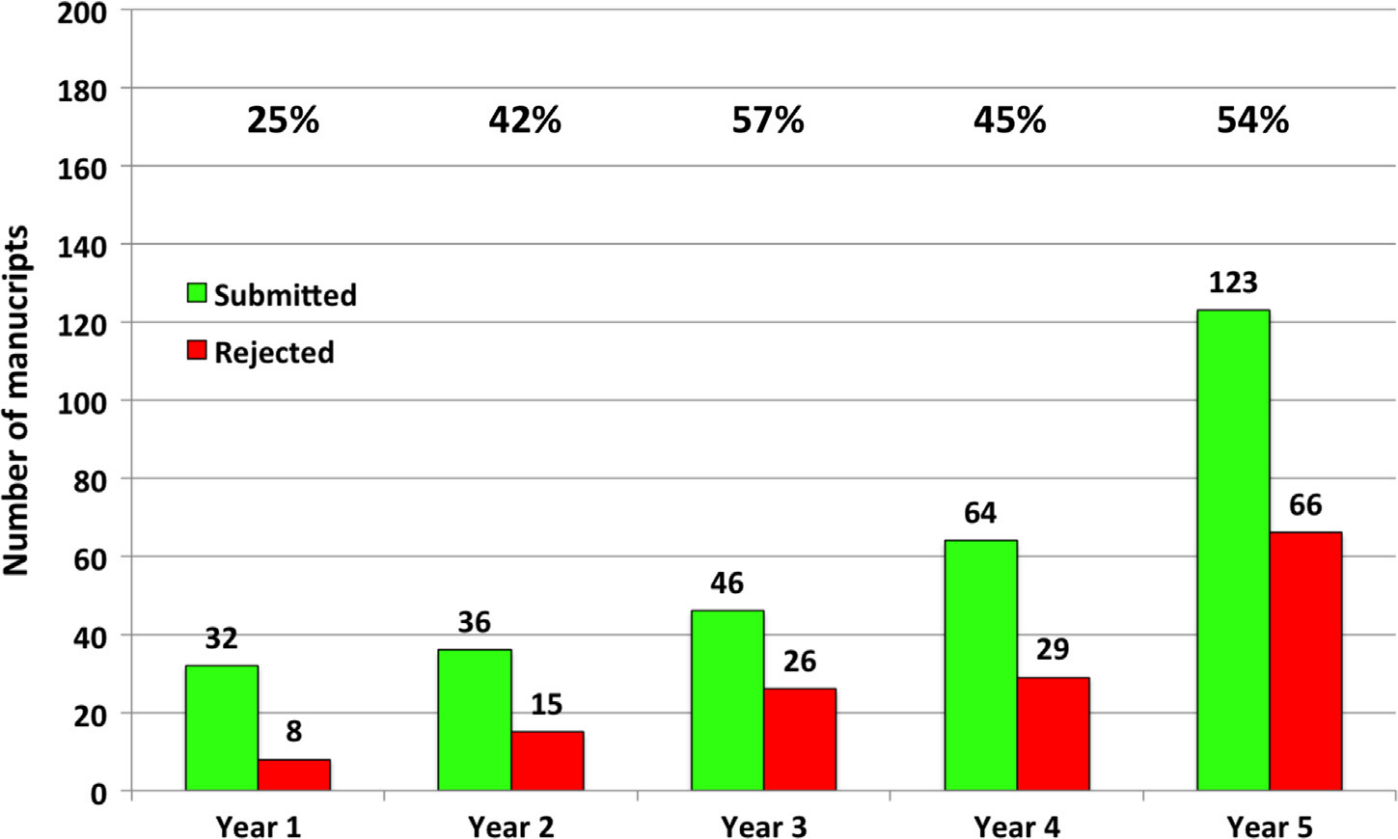
Number of manuscripts submitted and rejected in *Global Health Action*, excluding articles in special issues, editorials, and invited commentaries.

Submission to *GHA* has quadrupled from 51 manuscripts in the first 12 months to 190 manuscripts per annum (including articles in special issues and invited commentaries) in the fifth year. *GHA* pledges to ensure the highest possible quality of published manuscripts, and the 5-year rejection rate in *GHA* is 48% (ranging from 25% in the first year to 54% in the fifth year) as shown in Figure 3. The editorial team rejects poor quality manuscripts directly in-house. Two to three external reviewers, whose excellent work ensures high-quality manuscripts published in *GHA*, review each manuscript.

The percentage at the top of Figure 3 represents the proportion of manuscripts rejected in respective years. Only manuscripts for which a publication decision has been made are included in the analysis. At the time of writing this article, 34 manuscripts are under review as of 30th June 2013, and these are not included in the statistics for submitted manuscripts for Year 5.

### Time from submission to first decision

We aim to provide a quick decision to authors who submit their papers to *GHA*. Manuscripts that do not qualify for publication in *GHA* will be rejected within a few working days. We aim to provide first review-based decisions to authors within seven weeks following submission. The average time from submission to first decision decreased from about 55 days in the first year to 39 days in the fifth year.

### Time from decision to online publication

All of the manuscripts accepted for publication in *GHA* are published online immediately after the layout of the manuscript has been finalized. The average time from final acceptance to online publication has decreased from about 35 days in the first year to 27 days in the fifth year.

## Becoming a recognized and reliable source in global health literature

### View and download statistics

Over the years, *GHA* has earned inclusion in all major relevant indexing services, including MEDLINE, PubMed, CINAHL, Science Citation Index Expanded, Current Content, and HINARI. The journal is archived for posterity in PubMed Central, CLOCKSS and Portico, and also utilizes the LOCKSS system to create a distributed archiving system among participating libraries, and permitting those libraries to create permanent archives of the journal for purposes of preservation and restoration.

The journal reaches a global audience in 194 countries, the top 10 being USA, Sweden, India, UK, Canada, South Africa, Australia, Ethiopia, the Philippines, and Germany. From its launch until the first half of 2013, *GHA* articles have been downloaded 535,663 times (161,822 downloads in 2013 alone). There were 76,046 downloads in 2012 and 72,757 in the first half of 2013 from PubMed Central. *GHA* had about 54,713 visits during 2011, of which 38,526 were unique visitors. Two thirds of the traffic was from new visitors to the journal.

### Citation statistics

Harzing's Publish and Perish software (9) indicates that *GHA* has an H-Index of 19 as of mid-2013. A total of 19 papers published in *GHA* have thus each been cited 19 times or more and 583 times in total according to Google Scholar. These 19 papers have focused on chronic non-communicable diseases and their risk factors (10–17), climate change and health (18–21), ageing and wellbeing of the elderly population (22–24), migration and health (4), HIV and intervention program (25), maternal and other health issues (26, 27).

The data from the Web of Science show that a total of 616 citations have been accumulated from the 336 papers, including editorials and commentaries, published in *GHA* in the first five years. These citations appear in 364 scientific papers, of which 95 papers (26%) were published in *GHA*. The next 10 most common journals where *GHA* articles have been cited included: *BMC Public Health* (16 papers), *Industrial Health* (10 papers), *PLoS One* (eight papers), *Tropical Medicine and International Health* (eight papers), *International Journal of Epidemiology* (seven papers), *Journal of Rehabilitation Medicine* (seven papers), *Lancet* (seven papers), *International Journal of Circumpolar Health* (five papers), *Journal of Health Population and Nutrition* (four papers), and *PLoS Medicine* (four papers). In addition, *GHA* papers have also been cited in over 100 international peer-reviewed papers.

## Impact factors

*GHA* received its first impact factor in 2012. Thomson Reuters accepted *GHA* in late 2011 for inclusion in Current Contents/Social and Behavioural Sciences, Social Sciences Citation Index, and the Science Citation Index Expanded. The impact factor for 2011 (which is based on the number of citations during 2011 to articles published in the 2009 and 2010 volumes) was 1.267. The new impact factor for 2012 is 2.062. *GHA* is ranked 97/157 2011 and 57/158 2012 in the category of Public, Environmental & Occupational Health (3rd quartile) and *GHA*'s Scientific Journal Ranking in 2012 was 0.168, which is ranked within Q1 of the Health Policy category.

## Highlights from editorials and special issues

Several key editorials have been written by global health experts in the field of non-communicable diseases and climate change as well as experts from many key organizations such as WHO, the INDEPTH Network, and National Institutes of Health (Table 1).

**Table 1:** Selected keynote editorials published in *Global Health Action*, 2008–2013

Authors (Year)	Institutions	Titles
Stig Wall (2008) (1)	Umeå Centre for Global Health Research, Umeå, Sweden	Global Health Action – Fuelling a hands-on approach to global health challenges
Tim Evans, Carla AbouZahr (2008) (28)	World Health Organization, Geneva	INDEPTH@10: Celebrate the past and illuminate the future
Rainer Sauerborn, Tord Kjellstrom, Maria Nilsson (2009) (29)	Umeå Centre for Global Health Research, Umeå, Sweden	Health as a crucial driver for climate policy
Ruth Bonita (2009) (30)	University of Auckland, New Zealand	Strengthening non-communicable disease prevention through risk factor surveillance
Robert Beaglehole, Ruth Bonita (2010) (31)	University of Auckland, New Zealand	What is global health?
Richard Suzman (2010) (32)	National Institute of Ageing, National Institute of Health, US	The INDEPTH WHO-SAGE multicentre study on ageing, health and well-being among people aged 50 years and over in eight countries in Africa and Asia
Birgitta Evengård, Anthony McMichael (2011)	Umeå University, Sweden and Australian National University, Australia	Vulnerable populations in the Arctic
Luis Sambo (2012) (33)	Regional Director, WHO Regional Office for Africa	Towards global health equity: opportunities and threats

Since its inauguration through June 2013, *GHA* has published a total of 16 special issues or supplements, covering a wide range of research areas including climate change and disasters, non-communicable diseases, ageing, and mortality. Many of these represent cross-country research collaborations among researchers in LMICs, and many within the INDEPTH Network (www.indepth-network.org), which is a network of HDSS sites in Africa and Asia. Table 2 presents the topics and guest editors for selected special issues published during this period.

**Table 2:** Selected special issues published by *Global Health Action* during 2008–2012

Special issues	Guest editors	Foreword
‘Risk factors for chronic non-communicable disease’ (2009)	Prof. Ruth Bonita (University of Auckland, New Zealand)	Prof. Osman Sankoh (INDEPTH Network) and Dr. Ala Alwan (WHO, Geneva)
‘Climate change and global health: linking science with policy’ (2009)	Prof. Rainer Sauerborn (Heidelberg University, Germany), Prof. Tord Kjellstrom (Australian National University, Australia), Dr. Maria Nilsson (Umeå University, Sweden)	Dr. Maria Neira (WHO Headquarter, Switzerland)
‘Climate change impacts on working people’ (2010)	Dr. Maria Nilsson (Umeå University, Sweden), Prof. Tord Kjellstrom (Australian National University, Australia)	–
‘Analyses of mortality clustering at member HDSSs within the INDEPTH Network – an important public health issue’ (2010)	Prof. Heiko Becher (Heidelberg University, Germany)	Prof. Osman Sankoh (INDEPTH Network)
‘Growing older in Africa and Asia’ (2010)	Prof. Richard Suzman (National Institute of Aging, United State), Prof. Stephen Tollman and Dr. Kathy Kahn (Wits School of Public Health, South Africa), Dr. Nawi Ng (Umeå University, Sweden)	Prof. Osman Sankoh (INDEPTH Network) and Dr. Ties Boerma (WHO Headquarter, Switzerland)
‘Vulnerable populations in the Arctic’ (2011)	Prof. Birgitta Evengård (Umeå University, Sweden), Prof. Anthony McMichael (Australian National University, Australia)	Gustaf Lind (Ambassador to the Arctic, Swedish Ministry for Foreign Affairs)
‘CLIMO – Climate and Mortality’ (2012)	Dr. Joacim Rocklöv (Umeå University, Sweden), Prof. Rainer Sauerborn (Heidelberg University, Germany), Prof. Osman Sankoh (INDEPTH Network)	Dr. Abdul Rahman Lamin (UNESCO, Ghana)
‘Building new knowledge: Celebrating the Wits School of Public Health’ (2013)	Prof. Laetitia C. Rispel and Prof. Sharon Fonn (Wits School of Public Health, South Africa)	Prof. John Gear and Prof. William Pick (University of the Witwatersrand)
‘Public health in Vietnam: here's the data, where's the action?’ (2013)	Prof. Nguyen Duc Hinh and A/Prof. Hoang Van Minh (Hanoi Medical University)	Prof. Lars Weinehall (Umeå University), Prof. Nguyen Duc Hinh (Hanoi Medical University)
‘Global Health Beyond 2015’ (2013)	Prof. Peter Byass, Dr. Yulia Blomstedt, Prof. Stig Wall (Umeå University, Sweden) and Prof. Peter Friberg (University of Gothenburg)	–

## *GHA* – opportunities and way forward

*GHA* has become one of the leading journals in the public health field using the Open Access model. Since *GHA* was established in 2008, the number of Open Access public health journals has grown, including the latest arrival, *The Lancet Global Health* open access journal. In the call for papers written by the editors, Zoë Mullan and Richard Horton, they stressed the importance of “the work of researchers in low-income and middle-income countries who address urgent domestic concerns that don't always make it onto the front pages of general medical journals” (34). *GHA* has addressed this gap since its conception.

The rapid transition towards open access publishing is, on the one hand, an immense benefit to LMIC researchers, because they have free access to open access literature without needing to find resources for prohibitively expensive subscriptions. On the other hand, open access publishing cannot be totally free at all points in the process, and so support for paying publication fees is an important component for LMIC authors who rarely have either project or institutional funding for their work. Publication of an article in *GHA* incurs a relatively modest cost. However, to emphasize our commitment to extend an arena for publication in which developing settings can publish their research results, the publication fee may be waived for authors from institutions or projects unable to pay. *GHA* has received financial support from the Swedish Council for Working Life and Social Research during 2013–2015 to expand the number of waivers to highly qualified researchers from LMICs who do not have the resources for publication fees.

All articles published in *GHA* are freely accessible online immediately after they have been accepted for publication and can thereafter be linked, read, downloaded, stored, printed and used by anybody with a computer and access to the internet. The Open Access model offers additional multimedia benefits with links to videos, audios, full datasets and appendices, unlimited colour budgets, and interactive features, none of which the printed medium can provide.

Co-Action Publishing ensures that the best web technology supports the editorial team at Umeå University as well as the contributing authors and reviewers and thereby enhances the scholarly content of *GHA*. Open access serves the interests of all: readers, authors, teachers, students, libraries, universities, funding agencies, and ultimately governments and citizens. It increases the *visibility* of individual authors’ work; key resources are equally accessible to rich and poor; the mission of most universities to disseminate and share knowledge is facilitated, and funders (including governments) are given return on investment. Publishing open access directly contributes to the *democratization* of knowledge and helps reduce the digital divide between rich and poor nations.

We envision *GHA* as a leading journal in the global health area, with a mission to contribute to capacity building in global health research, especially in LMICs. We look forward to continue working with our reviewers and editorial board in the years to come to achieve the missions that *GHA* has delineated. We believe this collaboration will ensure that *GHA* will move from having taken first steps to taking major leaps in the near future.

**Global Health Action - Key Facts (May 2008–June 2013)**

- 505 manuscripts submitted, including 169 articles in special issues/supplements
- 301 independent manuscripts were submitted, 144 were rejected (5-year rejection rate of 48%)
- 336 papers published, 616 citations appear in 359 papers
- First impact factor: 1.267 in 2011
- Latest impact factor: 2.062 in 2012
- Rank 57/158 in the category “Public, Environmental & Occupational Health”
- H-index of 19 (using the Harzing's Publish and Perish software as of June 30, 2013).

*Nawi Ng* Managing Editor *Peter Byass* Deputy Editor *Stig Wall* Chief Editor for the Editorial Team of Global Health Action
